# Radiotherapy plus EGFR inhibitors: synergistic modalities

**DOI:** 10.1186/s41199-016-0020-y

**Published:** 2017-01-18

**Authors:** Paolo Bossi, Francesca Platini

**Affiliations:** 1 Head and Neck Medical Oncology, Fondazione IRCCS Istituto Nazionale dei Tumori, Via Venezian 1, 20133, Milan, Italy; 2 Medical Oncology Unit, University Hospital Maggiore della Carità, Novara, Italy

**Keywords:** Epidermal Growth Factor Receptor, Cetuximab, Gefitinib, Human Papilloma Virus, Erlotinib

## Abstract

Locally advanced (stage III or IV) squamous cell carcinoma of the head and neck (SCCHN) often requires multimodal treatment, consisting of a combination of surgery, radiation, and/or systemic therapy, namely chemotherapy or targeted agents. The expression of the epidermal growth factor receptor (EGFR) has been detected in more than 90% of all cases of SCCHN and has been correlated with decreased survival rates, resistance to radiotherapy, loco-regional treatment failure, and increased rates of distant metastases. This paper discusses several strategies aimed at targeting EGFR in combination with radiation. Until now, cetuximab, an anti-EGFR monoclonal antibody, is the only targeted agent that has been shown to improve overall survival in combination with radiation therapy. However, considering that there are multiple mechanisms of primary and acquired resistance to EGFR inhibitors, we focused on dissecting molecular pathways of EGFR inhibition to find alternative or complementary strategies for increasing tumour responsiveness. We suggest that the combination of treatments targeting the EGFR pathway and drugs aimed at increasing immune responses represent a promising approach that deserves to be further explored.

## Background

Squamous cell carcinoma of the head and neck (SCCHN) represents approximately 90% of all cancer arising in the head and neck area [1] and represents the sixth most common type of cancer, diagnosed in over 600,000 patients worldwide every year [2]. Prognostic factors in SCCHN are limited, except for the recognized positive role of human papilloma virus (HPV), mainly in association with oropharyngeal cancer. In this regard, SCCHN can be broadly divided into HPV-negative, more frequently associated with alcohol and tobacco consumption, and those where HPV is a causal factor [3]. Treatment choices are mainly based on the primary tumour site, TNM staging, and performance status. Advanced disease (stage III or IV) often requires multimodal treatment, consisting of a combination of surgery, radiation and/or chemotherapy [3]. When combined with radiation, systemic chemotherapy is most frequently based on platinum compounds, which have shown to yield the greatest benefits in combined treatment strategies [4].

On the quest for a better understanding of the molecular biology of SCCHN, researchers have focused on the introduction of targeted agents and on epidermal growth factor receptor (EGFR)-inhibitors in particular. In fact, the expression of the EGFR is detected in more than 90% of all cases of SCCHN and has been correlated with decreased survival rates, resistance to radiotherapy, loco-regional treatment failure, and increased rates of distant metastases [5]. Based on clinical relevance, the EGFR antibody cetuximab is the only monoclonal antibody (mab) to have been approved by the US Food and Drug Administration and the European Medical Agency, for use in combination with radiation in cases of locally advanced diseases and added to platinum-based chemotherapy in cases of recurrent-metastatic SCCHN [6, 7].

The aim of this paper is to review clinical trials and translational studies that pursue therapeutic approaches based on radiotherapy plus EGFR inhibition and illustrate their benefits, suggesting that further work should be done in this direction.

### Molecular mechanisms of EGFR blockade plus radiation and resistance pathways

Since EGFR is an established target in SCCHN treatment, several molecular mechanisms may explain the synergistic effect of EGFR-targeted therapies and radiation. In fact, the combination of EGFR blockade and radiation exploits three distinct roles that the EGFR pathway plays in cancer progression: enhanced cell proliferation, the activation of pro-survival pathways, and DNA repair [8]. What follows is a brief explanation of these functions and of the mechanisms of resistance to anti-EGFR therapies.

#### Enhanced cell proliferation

The accelerated proliferation of tumour cells during radiotherapy is one reason for loco-regional therapies failure. Shortening overall treatment time makes tumour control more likely, so that tumour cells repopulation between radiotherapy fractions could be more difficult. The accelerated proliferation rate could result from the activation of EGFR in response to ionising radiation, which would indicate that EGFR-related signalling is involved in the proliferative response to radiotherapy, thereby enhancing survival probabilities [9].

#### Activation of pro-survival pathways

Resistance to radiotherapy could also be caused by EGFR downstream effectors known to activate pro-survival pathways.

In this regard, the role of cetuximab as a “radiosensitizer” could be explained by its capacity to partially inhibit STAT-3 (the signal transducer and activator of transcription 3), an apoptosis protection mediator.

Focusing specifically on SCCHN, prior research has found over-expression of mTOR and genomic alteration in the PI3K/AKT signalling pathway to be associated with decreased overall survival rates [10]. In addition, pAKT expression has been proven to be a putative biomarker predictor of response to cetuximab-based chemotherapy [11]. According to a 2015 study by Schuettler et al., irradiation induces the phosphorylation of AKT, p38 MAPK, and ERK [12]. The combined activation of these pathways has been proven to cause inactivation of GSK3β kinase, resulting in enhanced tumour cell migration. Furthermore, as shown by Mishra et al., inhibition of GSK3β activates wnt-/β-catenin signalling, which has been linked to enhanced cell migration in oral squamous carcinoma cell lines [13]. This means that the specific inhibition of even one of the EGFR’s downstream pathways is sufficient to restore GSK3β activity and reduce radiation-induced migration in SCCHN.

#### DNA repair

EGFR is known as a cell surface receptor, but resistance to radiation therapy has also been related to nuclear EGFR expression, an isoform that plays an important role in gene expression (such as cyclin D1, COX-2, c-Myc and aurora kinase A) and in DNA repair [14]. Radiation promotes the internalization and transport of EGFR by caveolin-1, leading to the activation of DNA-PK (a vital kinase for joining repair) in response to DNA damage. In this respect, researchers have shown that inhibition of EGFR with cetuximab attenuates EGFR nuclear import and suppresses DNA-PK activity [8].

### Promising trials and next steps to overcome resistance

In a recent study, Bonner and colleagues explored the combined *dual inhibition of EGFR and JAK–STAT-3* with and without radiation in human SCCHN cell lines. They found that combining cetuximab and radiation therapy with adjunct treatment that targets the JAK1 enhances the anti-proliferative, apoptotic, and radio-sensitizing effects of radiation, causing an increase of unrepaired radiation-induced DNA double-strand breaks when cells are exposed to both drugs [15].

In addition, clinical evidence also shows that the EGFR blockade activates the MET pathway (as a compensatory mechanism, thus causing resistance to EGFR inhibitors. This further supports the strategy that aimed at *dual blocking of HGF/MET and EGFR* pathways [16].

Another mechanism that supports the innovative strategy of integrating *dual blocking* with a combined therapeutic approach is the cross-talk between *EGFR and VEGF* survival pathways. Several clinical studies have yielded positive results when using a combination of bevacizumab, cetuximab, and chemotherapy in addition to radiation or when adding bevacizumab to the combination of erlotinib, chemotherapy, and radiation [17–19]. Further exploration of these combined strategies and their safety profiles thus seems like a promising direction to pursue.

Moreover, there is also evidence of the Hedgehog pathway being relevant to a novel cetuximab resistance mechanism involving *epithelial to mesenchymal transition* (EMT). Specifically, a recent phase I trial has shown that cetuximab in combination with IPI-926, a hedgehog pathway inhibitor, yields anti-tumour activity with well-tolerated toxicities [20].

### Inhibition of EGFR: monoclonal antibodies and tyrosine kinase inhibitors

There are two main ways to inhibit EGFR signalling pathways: monoclonal antibodies targeting EGFR, which directly interfere with the ligand receptor, and tyrosine kinase inhibitors, which block the intracellular domain with tyrosine kinase activity [1].

#### Monoclonal antibodies

When speaking of SCCHN, the most investigated monoclonal antibodies that specifically bind to EGFR are cetuximab, panitumumab, nimotuzumab, and zalutumumab. Table 1 summarizes the main trials in locoregionally advanced SCCHN.

**Table 1:** Main trials with monoclonal antibodies associated with radiotherapy in locally advanced SCCHN

Study	Treatment	Efficacy				Toxicities		
		LRC	PFS	OS	RR	G3-4 dermatitis	G3-4 mucositis	G3-4 dysphagia
Bonner et al. [6, 21]	RT			5-yr 36.4%		18%	52%	
	RT + cetuximab			5-yr 45.6%		23%	56%	
Fury MG et al. [17]	cet + bevacizumab + CDDP + RT		2-yr 88.5%	2-yr 92.8%			3%	3%
Lefebvre JL et al. [22]	TPF → RT + CDDP vs			92%		26%	43%	
	TPF → RT + cet			89%		57%	43%	
Mesia R et al. [25]	RT + cet vs	1-yr 47%					2%	
	RT + cet + 12 weeks maint	1-yr 59%					7%	
		2-yr similar						
Egloff AM et al. [47]	Cet + CDDP +		2-yr 47%	2-yr 66%	66.7%	26%	55%	46%
	RT + cet maint							
Ang KK et al. [26]	RT + CDDP vs		3-yr 61.2%	3-yr 72.9%			33%	
	RT + CDDP + cet		3-yr 58.9%	3-yr 75.8%			43%	
Mesia R et al. [28]	RT + CDDP vs	2-yr 68%					24%	27%
	RT + CDDP + PMab	2-yr 61%					55%	40%
Giralt J et al. [29]	RT + PMab vs	2-yr 51%					40%	42%
	RT + CDDP	2-yr 61%					32%	40%
Siu LL et al. [30]	RT + CDDP vs		2-yr 73%	2-yr 85%				
	RT + Pmab		2-yr 76%	2-yr 88%				
Reddy BK et al. [31]	RT + CDDP + NMab			5-yr 57%			11%	4%
	CDDP + RT			5-yr 26%			5%	1%
	RT + NMab			5-yr 39%			10%	2%
	RT			5-yr 26%			16%	
Eriksen JG et al. [33]	RT+ CDDP + nimo + ZMab	4-yr 71%				29%		
	Vs RT + CDDP + nimo	4-yr 73%						

*Abbreviations*: *Cet* cetuximab, *PMab* panitumumab, *NMab* nimotuzumab, *ZMab* zalutumumab, *nimo* nimorazole, *CDDP* cisplatin, *CHT* chemotherapy, *RT* radiotherapy, *LRC* locoregional control, *OS* Overall Survival, *RR* response rate, *PFS* Progression-Free Survival, *RR* response rate, *G* grade

Cetuximab was the first monoclonal antibody to be investigated and until now has been deployed in various treatment strategies based on radiation therapy (which we discuss in detail below).

The pivotal study by Bonner et al., already mentioned above and discussed in several journals, showed that in cases of locoregionally advanced SCCHN, patients treated with a combination of cetuximab plus radiotherapy had an advantage in 5-year overall survival (OS), compared to those threated by radiation alone (5-year OS 45.6% vs. 36.4%). In addition, overall survival improved significantly if the patient developed rashes of grade-2 severity [6, 21]. Cetuximab plus radiation has also been investigated after induction chemotherapy (docetaxel, cisplatin and 5-fluorouracil, TPF).

For instance, the Tremplin study, which explored a new combination strategy for organ preservation in cases of laryngeal and hypopharyngeal cancer, involved TPF followed by radiation in combination with either cisplatin or cetuximab. The study proved that there was no difference in disease control and in overall survival between the two combinations. The only differences found were that cisplatin yielded higher local control and that only the cetuximab treated group required salvage surgery [22]. A Spanish trial investigated the same strategy in cases of locally advanced SCCHN, most of which HPV negative. The results showed a trend of better PFS (HR 1.20) and OS (HR 1.17) when using cisplatin in addition to radiation after induction TPF [23]. As expected, the two drugs yielded a very different toxicity profile: cetuximab was associated with more mucosal and skin toxicity and cisplatin with greater nephrotoxicity.

Another important trial study, known as GORTEC 2007–02, compared the use of chemoradiation (with carboplatin and 5FU) in concurrence with induction TPF followed by radiation with cetuximab, in locally advanced clinical stage N2b-N3 SCCHN and found no difference between the two test groups. This suggests that concurrent chemoradiation (even if not performed with cisplatin) remains the best treatment also with a high burden of nodal disease [24].

Another randomized phase II trial, instead, explored an adjuvant treatment strategy that consisted of administering cetuximab for twelve weeks after use in combination with radiotherapy. Despite favourable results for locoregional control after 1 year, no difference was observed at the 2-year stage [25].

There is also a number of trials that investigate the association of cetuximab to cisplatin and radiation. The largest of these preliminary studies, the RTOG 0522, shows that cetuximab plus cisplatin, in comparison to cisplatin alone, yields increased acute toxicities, more frequent radiotherapy interruption, and no survival benefits [26]. Recently, the GORTEC 2007–01 phase III randomized trial showed that the addition of concomitant chemotherapy to cetuximab-based radiotherapy markedly improved progression-free survival and locoregional control, with a non-significant gain in survival [27]. This study targeted patients with limited locally advanced disease (N0-N2a), mainly HPV negative (65% of the patients had oropharyngeal cancer, but only 20% of them were p16 positive). Results showed that in this population the additional use of chemotherapy produced better results than treatment based just on cetuximab plus radiation.

Moving on to other monoclonal antibodies, an important reference is the set of CONCERT trials (CONcomitant Chemotherapy and/or EGFR inhibition with Radiation Therapy), which investigated the use of panitumumab in addition or in substitution to cisplatin-based chemotherapy and in combination with radiation, for the treatment of locally advanced SCCHN. For each trial, the results showed that this strategy does not yield significant benefits [28, 29]. Specifically, the CONCERT-1 trial concluded that the addition of panitumumab to chemoradiation not only did not yield any superior efficacy, but led to an increase in acute toxicity. Instead, the CONCERT-2 trial concluded that panitumumab does not constitute a viable substitute to cisplatin, in light of an inferior primary endpoint of locoregional control at the 2-year stage (51% with panitumumab vs. 61% with cisplatin). In a recent study, instead, panitumumab was added as radiosensitizer to accelerated fractionation radiation and compared to standard fractionation radiation in combination with cisplatin [30]. The strategy resulted in a higher than expected survival probability for the entire group, but this is explained by the fact that the test population was mainly constituted by patients affected by oropharyngeal cancer (81%), most of whom were p16 positive. Aside from this, the study showed that treatment with panitumumab is not more effective than chemotherapy, although the altered fractionation scheme did not allow for a formal test of non-inferiority. Overall, the data clearly demonstrates that panitumumab cannot and should not replace cisplatin in combined treatment with radiotherapy.

Finally, as regards nimotuzumab, in a phase-II trial the use of the monoclonal antibody in addition to chemoradiation or radiation seemed to provide long-term survival benefits [31]. Another phase II trial enrolled 106 patients with unresectable SCCHN and randomized them for treatment with either radiotherapy alone or with radiotherapy in combination with nimotuzumab and showed a significant complete response rate improvement in the group of patients treated with nimotuzumab [32]. Instead, in the case of zalutumumab, the Danish Head and Neck Cancer Group (DAHANCA) inquired whether the addition of the monoclonal antibody during radiotherapy could improve outcome in patients with locally advanced SCCHN and found no difference in locoregional control [33].

#### Tyrosine kinase inhibitors

Several EGFR tyrosine kinase inhibitors (TKI), such as lapatinib, gefitinib and erlotinib, have been investigated in SCCHN concurrently with radiation, as shown in Table 2. According to a phase III trial, there are no survival benefits associated with lapatinib, a small-molecule inhibitor of EGFR and of the human epidermal growth factor receptor 2 (HER2, ErbB2), neither when paired with chemoradiation nor when used as a maintenance monotherapy in patients with high-risk surgically-treated SCCHN [34].

**Table 2:** Main trials with tyrosine kinase inhibitors associated with radiotherapy in locally advanced SCCHN

Study	Treatment	Efficacy					Toxicities		
		LRC	PFS	OS	RR	LDCR	G3-4 dermatitis	G3-4 mucositis	G3-4 dysphagia
Harrington K et al. [34]	RT + CDDP vs			5-yr 57.3%					
	RT + CDDP+ lapatinib			5-yr 56.6%					
Martins RG et al. [35]	CDDP + RT vs		No difference		40%		2%		
	CDDP + RT+ erlotinib				52%		13%		
Hainsworth JD et al. [19]	CBDCA + Tax + 5FU + beva → RT+ Tax + beva + erlotinib		3-yr 71%	3-yr 82%				88%	
Yoo DS et al. [18]	Beva + erlotinib + CDDP + RT	3-yr 85%	3-yr 82%	3-yr 86%				48%	28%
Rao K et al. [36]	RT+ intra-arterial CDDP + erlotinib			1-yr 63%					
Gregoire V et al. [37]	CDDP + RT vs					2-yr 33.6%		36%	13%
	CDDP + RT + gefitinib +/− maint gefitinib					2-yr 32.7%		47%	5%
Cohen EE et al. [38]	FU + hydroxyurea + RT + gefitinib + 2-years maint gefitinib		4-yr 72%	4-yr 74%			33%	55%	
Hainsworth JD et al. [48]	CBDCA + Doc + 5FU + gefitinib followed by RT + Doc + gefitinib and 2-years gefitinib		3-yr 41%	3-yr 54%				27%	
Rodriguez CP et al. [49]	RT + CDDP + 5FU + gefitinib +2-years gefitinib			3-yr 71%				87%	

*Abbreviations*: *CBDCA* carboplatin, *Tax* paclitaxel, *Doc* docetaxel, *5FU* 5-fluorouracil, *CDDP* cisplatin, *beva* bevacizumab, *RT* radiotherapy, *LDCR* local disease control rate, *LRC* locoregional control, *OS* Overall Survival, *DFS* Disease Free Survival, *PFS* Progression-Free Survival, *RR* response rate, *G* grade, *maint* maintenance

Another phase II trial by Martins et al. randomly selected 204 patients to receive radiotherapy plus cisplatin, with or without the addition of erlotinib. Even if well tolerated, erlotinib failed to produce a significant improvement in both the complete response rate and in progression-free survival [35]. Hainsworth et al. evaluated the feasibility and efficacy of adding both bevacizumab and erlotinib to concurrent chemoradiation (CRT) as a first-line treatment in locally advanced SCCHN. The 3-year progression-free survival rate and the overall survival rate for the entire group were respectively 71% and 85% and the most frequent severe toxicity was grade 3/4 mucosal toxicity [19]. Several other studies investigated the safety and efficacy of erlotinib combined with concurrent chemoradiation, but their sample size was too small to allow for any significant conclusions [18, 36].

There is also evidence against potential benefits to be gained from gefitinib. Specifically, a randomized phase II trial based on 226 subjects tested the differences between treating patients with gefitinib 250 mg/day, gefitinib 500 mg/day, or placebo. The study was structured in two phases: a concomitant phase (gefitinib or placebo with chemoradiation) and a maintenance phase (gefitinib or placebo alone). The investigators concluded that treatment with gefitinib did not improve 2-year loco-regional control compared to placebo, neither when given concomitantly with chemoradiation nor as maintenance therapy [37]. Another phase II study investigated the feasibility of administering gefitinib in concomitance with chemoradiation and then alone as maintenance therapy for two years. Gefitinib proved to be well tolerated in the adjuvant phase, but associated with a high incidence of treatment-related death during the first concurrent phase [38].

Finally, the LUX-Head and Neck 2 trial, a phase III study that is still in progress, might be able to say a definitive word on the role of afatinib, an irreversible ERbB-family blocker. Specifically, the study aims to assess the use of afatinib versus placebo as an adjuvant treatment following concurrent chemoradiation in primary unresected locoregionally-advanced SCCHN, with disease-free survival as a primary endpoint [39].

### The role of EGFR inhibition in HPV-positive versus HPV-negative SCCHN

HPV-positive types of SCCHN are driven by the integration of HPV DNA into the host genome and the activation of specific and consistent molecular regulators, including p16^INK4^, representing a distinct SCCHN entity [3]. Analyses of 279 cases of SCCHN by The Cancer Genome Atlas identified p53 mutations in 84% of HPV-negative tumours and only in 3% of HPV-positive tumours, supporting the thesis that smoking and alcohol related cases of SCCHN are associated with near-universal loss of p53 function through its mutation and with CDKN2A inactivation. On the other hand, HPV-positive cancers cause p53 degradation through the binding of the E6 protein with the ubiquitin ligase E6-associated protein (E6AP) to the p53 of host cells. However, this mechanism of p53 alteration explains only in part the different chemo- and radio-sensitivity of HPV-positive versus HPV-negative cancers.

Further investigation in this direction is represented by a set of preclinical studies that tested interesting hypothesis for radio-sensitization of HPV-positive cancer cells. Specifically, Ziemann et al. showed that cell cycle deregulation and the down-regulation of HPV E6 and E7 proteins serve to promote the enhanced sensitivity of HPV+ SCCHN cells to simultaneous radio-chemotherapy [40]. In this regard, it is worth noting that cetuximab has been shown to inhibit the growth of E6- and E7-expressing tumours grafted in NOD-SCID mice, thus offering further evidence of the combined effect of radiotherapy and anti-EGFR treatment in HPV-positive cancers [41].

Other significant findings from a clinical point of view are associated to a study by Rosenthal et al. that evaluated the association between p16 and HPV expression by carrying out a retrospective analysis of the phase III IMCL-9815 study, which compared the effects of combined treatment by radiotherapy plus cetuximab versus treatment by radiotherapy alone. The study confirmed the prognostic role of p16 in both groups of patients, but was not able to assess p16 as a predictive factor for response to cetuximab. However, it should be noted that the magnitude of benefits obtained by the adjunct use of cetuximab were higher in p16-positive tumours than to p16-negative ones [42].

#### Ongoing trials

There are also several trials that are investigating the possibility of de-escalating treatment intensity among patients affected by HPV-positive oropharyngeal cancer, by using cetuximab as a radiosensitizer and comparing the efficacy of cetuximab versus cisplatin concurrently with radiation (RTOG 1016, De-ESCALaTE and TROG 12.01). The results of these trials will be especially important since, now, there are no biological markers in HPV-positive SCCHN known to have a predictive value for response to EGFR inhibition.

### New perspectives: anti-EGFR + immunotherapy

The immunotherapeutic approach is gaining more and more consensus in the treatment of cancer. As regards SCCHN patients in particular, there are several ongoing trials that are producing encouraging results so far, with a response rate of about 20% in the heavily pre-treated setting of second-line treatment for relapsed-metastatic patients [43–45] and long-lasting responses in a number of cases, something that was extremely rare in previous trials involving treatment with chemotherapy and cetuximab [46].

This evidence is of importance, since the SCCHN population is known to have reduced immunoreactivity towards cancer, as proven by low absolute lymphocyte counts, an impaired natural killer-cell pool, a poor antigen-presenting function, the impairment of tumour-infiltrating T lymphocytes, and suppressive regulatory T cells that secrete suppressive cytokines such as TGF-β and IL-10 [3–55]. Some researchers suggest that the lack of immunological control in SCCHN may also be driven by the expression of immune-inhibitory checkpoints, mainly the cytotoxic T-lymphocyte-associated antigen 4 (CTLA-4) and the programmed cell death protein 1 (PD-1), which normally regulate the ongoing immune response to prevent damage to healthy tissues [56–58]. Furthermore, SCCHN associated with alcohol and tobacco consumption is characterized by a high number of gene mutations [59] and, as we know, the mutational load is one of the genetic cancer factor influencing the possible restoration of an effective immune response. In light of these premises, SCCHN seems like good candidate for studies aimed at investigating the pursuit of immunotherapeutic strategies in combination with existing therapies of known value [60].

Returning to the line of inquiry focused on cetuximab, since the antibody works not only by blocking EGFR-related downstream pathways, but also by mediating the antibody-dependent cellular cytotoxicity (ADCC) of NK cells [61] as well as complement-mediated cytotoxicity [62] and adaptive immunity [63], it may be worthwhile to invest in the pursuit of EGFR therapies that also target secondary immune responses [64].

Moreover, since the immune response to cetuximab alone is limited, as witnessed by the limited rate of long-term responders, we suggest that clinical research should focus especially on combined treatments aimed at overcoming the immune evasion to anti-EGFR therapy. In SCCHN, the constitutive activation of STAT3 is responsible for tumour-immune evasion, producing immunosuppressive mediators and creating an immune tolerant microenvironment [65], In light of this, EGFR-independent STAT3 activation could contribute to a reduced response to cetuximab. If this were the case, the blockade of both targets might constitute a new therapeutic strategy [66]. Moreover, as shown by Pollack et al., EGFR-blockers may overcome the inhibitory effect of EGFR-signalling by increasing MHC expression [67]. We thus also suggest that the overall efficacy of EGFR inhibitor-targeted therapy in SCCHN patients could be enhanced by the addition of T-cell-based immunotherapy. A study by Kumai et al. offers promising evidence in this respect, showing that the EGFR875–889 peptide induced effective anti-tumor CD4 T-cell responses against cancer that expressed EGFR. The authors thus suggest that the peptide could serve as an effective cross-reacting epitope, inducing responses to other HER family members and to the c-Met antigen [68]. Another encouraging phase Ib study aimed at evaluating the effectiveness of using cetuximab with motolimod, a small-molecule TLR-8 agonist that activates myeloid dendritic cells, monocytes, and natural killer cells. Preliminary results show a response rate of 17% and a disease control rate of 50% in SCCHN patients [69]. However, the results of a recently presented randomized trial showed that adding motolimod to standard platinum, 5-fluorouracil and cetuximab therapy for metastatic SCCHN resulted in no benefit in OS and PFS [70]. Finally, another reason for pursuing combined approaches is that cetuximab monotherapy promotes Tregs expansion, which in turn increases immune-suppression in the tumoral microenvironment, particularly towards NK cell activity [71]. The expression of CTLA-4 on Tregs thus motivates a new therapeutic approach based on a combination of cetuximab and ipilimumab, aimed at increasing immune response against the tumour [72]. This combined treatment is currently undergoing phase Ib testing along with radiation, in stage III-IV SCCHN (NCT01935921).

## Conclusions

The use of radiation therapy in combination with cetuximab, an anti-EGFR monoclonal antibody, has been shown to improve overall survival in SCCHN patients. However, since clinical studies have shown that not all tumours are sensitive to EGFR inhibition and that others may develop acquired resistance, we suggest that a better understanding of the molecular mechanisms involved in EGFR-resistance is crucial to developing optimal therapeutic approaches. We believe that clinical research should focus on the use of combination or sequential targeted therapies that involve strategies aimed at enhancing immune response.

## Abbreviation

ADCC: Antibody-dependent cellular cytotoxicity
CRT: Chemoradiation
CTLA-4: Cytotoxic T-lymphocyte-associated antigen 4
EGFR: Epidermal growth factor receptor
EMT: Epithelial to mesenchymal transition
HER2: Human epidermal growth factor receptor 2
HPV: Human papilloma virus
OS: Overall survival
PD-1: Programmed cell death protein 1
SCCHN: Squamous cell carcinoma of head and neck
TKI: Tyrosine kinase inhibitors
TPF: Docetaxel, cisplatin and 5-fluorouracil.
